# Pneumomediastinum and Pneumopericardium in Rapidly Progressive Interstitial Lung Disease Secondary to Anti‐MDA5 Dermatomyositis

**DOI:** 10.1002/ccr3.71304

**Published:** 2025-10-16

**Authors:** Thomas Bond, Karan Kanani, Mili Pansuria, Kehinde Sunmboye

**Affiliations:** ^1^ Rheumatology University Hospitals of Leicester NHS Trust Leicester UK; ^2^ College of Life Sciences University of Leicester Leicester UK

**Keywords:** anti‐MDA5, dermatomyositis, interstitial lung disease, myositis, pneumomediastinum, pneumopericardium, pneumothorax

## Abstract

Anti‐melanoma differentiation associated protein 5 antibody positive dermatomyositis (anti‐MDA5 dermatomyositis) is characterized by rapidly progressive interstitial lung disease and skin involvement with a paucity of muscle involvement. Morbidity and mortality primarily result from lung involvement, which often responds poorly to immunosuppressive treatment. Therapy for Anti‐MDA5 dermatomyositis consists of high‐dose corticosteroid therapy in combination with other immunosuppressive therapies. Recognizing and treating anti‐MDA5 dermatomyositis quickly is imperative to avoid worse outcomes. We report a 74‐year‐old woman who initially presented with a 2‐month history of an erythematous rash over the right greater trochanter, unresponsive to oral flucloxacillin. She later developed acute shortness of breath, haemoptysis, and characteristic dermatomyositis features, including Gottron's papules and a shawl rash. CT pulmonary angiography showed bilateral basal peripheral and perilobular consolidations, with appearances compatible with an organizing pneumonia pattern; no pulmonary embolism was identified. Although her creatinine kinase (CK) was normal, an MRI of both her thighs was requested to assess for muscle inflammation in spite of normal CK levels. This revealed bilateral proximal muscle and subcutaneous oedema. She was diagnosed with dermatomyositis and started on high‐dose prednisolone 40 mg daily while awaiting myositis antibody results and skin biopsy. Three weeks later, she represented with worsening dyspnoea, dysphagia, and chest pain. Repeat CTPA showed new pneumothorax, pneumomediastinum, and pneumopericardium. She received intravenous methylprednisolone followed by intravenous immunoglobulins, but her condition deteriorated, and she died shortly thereafter. Postmortem results confirmed anti‐MDA5 antibody positivity. Anti‐MDA5 dermatomyositis is an important differential in patients with new onset interstitial lung disease, pneumomediastinum, and pneumopericardium due to its rapidly progressive nature. Thorough history and examination for skin manifestations of anti‐MDA5 dermatomyositis is crucial so that a diagnosis can be made promptly and treatment started at the earliest opportunity to avoid development of fatal disease‐related complications and mortality.

## Graphical abstract

Anti‐MDA5 dermatomyositis presenting with pneumomediastinum and pneumopericardium in the setting of rapidly progressive interstitial lung disease. These air‐leak complications signal severe, uncontrolled pulmonary inflammation. Early recognition of cutaneous clues and prompt, multidisciplinary escalation of immunosuppression are critical to improving survival.

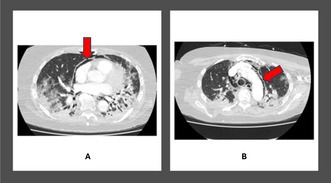

**Summary**

- Anti‐MDA5 dermatomyositis can present with subtle cutaneous signs and rapidly progressive interstitial lung disease.
- Early recognition is vital, as complications like pneumomediastinum, pneumothorax, and pneumopericardium indicate severe disease.
- Delay in diagnosis or treatment can lead to rapid deterioration and death despite aggressive immunosuppression.
- Prompt, multidisciplinary intervention is essential.

## 1 Introduction

Anti‐melanoma differentiation associated protein 5 antibody positive dermatomyositis (anti‐MDA5 dermatomyositis) is a subtype of dermatomyositis with high mortality. The paucity of muscle involvement can lead to a delay in diagnosis, worsening outcomes for this cohort of patients. Anti‐MDA5 dermatomyositis presents with skin involvement which can include skin ulceration along with rapidly progressive interstitial lung disease (RP‐ILD) [1]. RP‐ILD is the predominant cause of morbidity and mortality and often responds poorly to treatment in this cohort of patients [2]. Anti‐MDA5 dermatomyositis can be further complicated by pneumothorax and pneumomediastinum if disease control is not optimized quickly [3]. In addition, patients with anti‐MDA5 dermatomyositis are more vulnerable to infections, increasing the risk associated with the use of multiple immunosuppressive therapies in these patients [3]. Treatment consists of the use of high‐dose oral or intravenous corticosteroid therapy in combination with other immunosuppressants such as calcineurin inhibitors (such as ciclosporin or tacrolimus), mycophenolate cyclophosphamide, or rituximab [2]. Here we report a case of anti‐MDA5 dermatomyositis complicated by RP‐ILD with associated pneumomediastinum, pneumopericardium, and pneumothorax.

## 2 Case History/Examination

The 74‐year‐old lady initially presented to her general practitioner (GP) in September 2024 with a 2‐month history of erythematous rash and swelling on the lateral aspect of her right hip. She was diagnosed with cellulitis by her GP. After an initial course of oral flucloxacillin 1 g QDS for 7 days was not effective, an ultrasound of the area showed subcutaneous oedema but no underlying cutaneous collection. She was referred to the hospital for intravenous treatment and dermatology review. After a course of IV teicoplanin, she was prescribed dermovate cream and an extended course of doxycycline and then discharged home.

**FIGURE 1:**
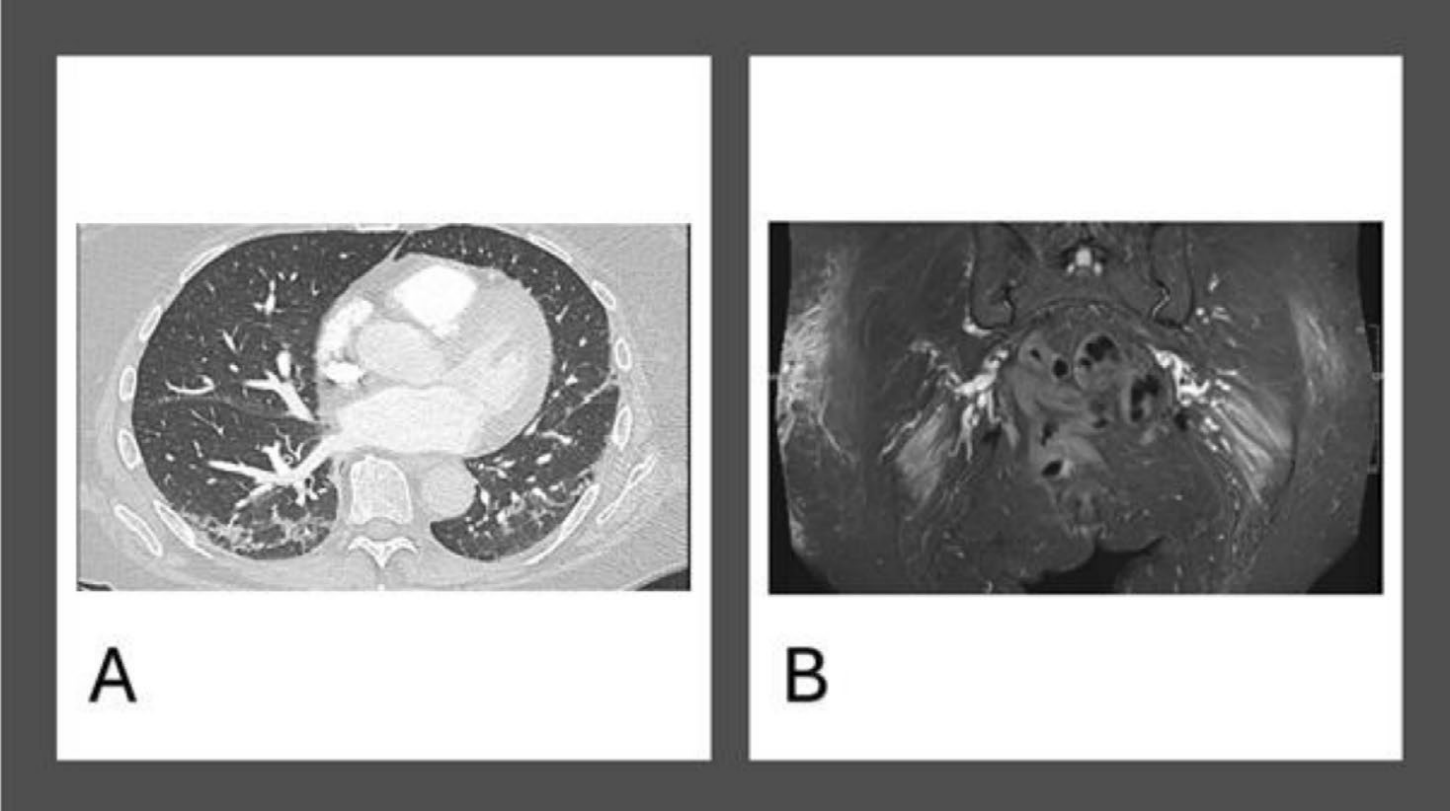
(A) CT chest imaging of the patient showing bilateral basal peripheral and perilobular consolidations. (B) An MRI of both femurs demonstrating diffuse asymmetric increased fluid signal on STIR involving the gluteus maximus muscles bilaterally, slightly more on the left and subcutaneous oedema focused on the area of her Holster's sign.

She represented to the emergency department a month later with acute shortness of breath and haemoptysis, and was admitted under the hospital respiratory team. She reported unintentionally losing 9 kg in weight over the last 8 weeks. Her lateral thigh rash was worse and oozing pus, and she had developed new skin lesions on her hands and anterior chest. She also reported night sweats and fevers along with generalized weakness and malaise. She had a total body CT to evaluate for any mitotic lesions and a CTPA to exclude pulmonary embolism. The CT scan did not show a blood clot but showed bilateral basal peripheral and perilobular consolidations (Figure 1A).

No suspicious signs of malignancy were found on total body CT. A further in‐hospital review by the Rheumatology team confirmed that Gottron's papules, a V‐sign, and shawl sign were present on examination on her hands, anterior chest and back respectively. Muscle power in all limbs were not affected. She had tumor markers to exclude significant elevations which may often highlight any underlying age‐related cancers. This showed only a mildly elevated carcinoembryonic antigen (CEA) of 9 IU/mL. An MRI of both femurs demonstrated bilateral proximal diffuse subcutaneous oedema (Figure 1B). A biopsy of the right hip lesion was performed and a myositis antibody screen was sent. High‐dose oral prednisolone therapy at 40 mg OD, weaning by 10 mg every 2 weeks, was commenced. Outpatient rheumatology review was arranged to initiate disease‐modifying therapy. She was discharged 2 weeks after initial admission to hospital.

### 2.1 Differential Diagnosis

Whilst an infected cutaneous rash was the initial main differential, the progression of the patient's rash, with a lack of response to oral and intravenous flucloxacillin therapy, was more indicative of an autoimmune process. The normal CK implied an amyotrophic dermatomyositis. In patients where dermatomyositis is suspected, it is important to rule out malignancy, which is why CT scans and tumor markers were requested in this patient. Myositis antibody screening and a skin biopsy were also performed to support the diagnosis and characterize the nature of her inflammatory myositis. In the meantime, high‐dose corticosteroids were started to treat the underlying inflammatory process.

### 2.2 Outcome

**FIGURE 2:**
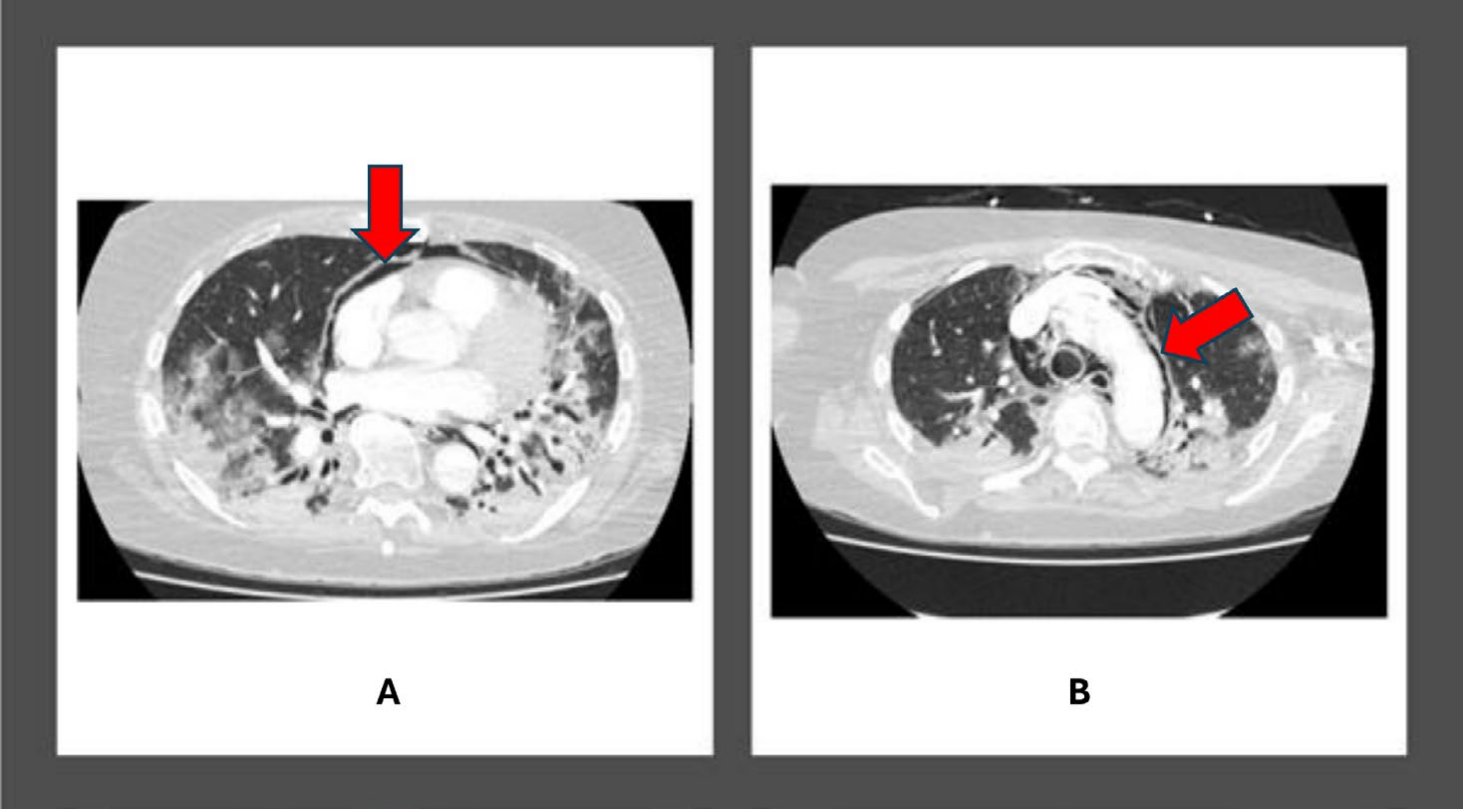
CTPA demonstrating significant disease progression with bilateral basal peripheral and perilobular consolidations. (A and B) with red arrows showing pneumopericardium (A) and pneumomediastinum (B).

**FIGURE 3:**
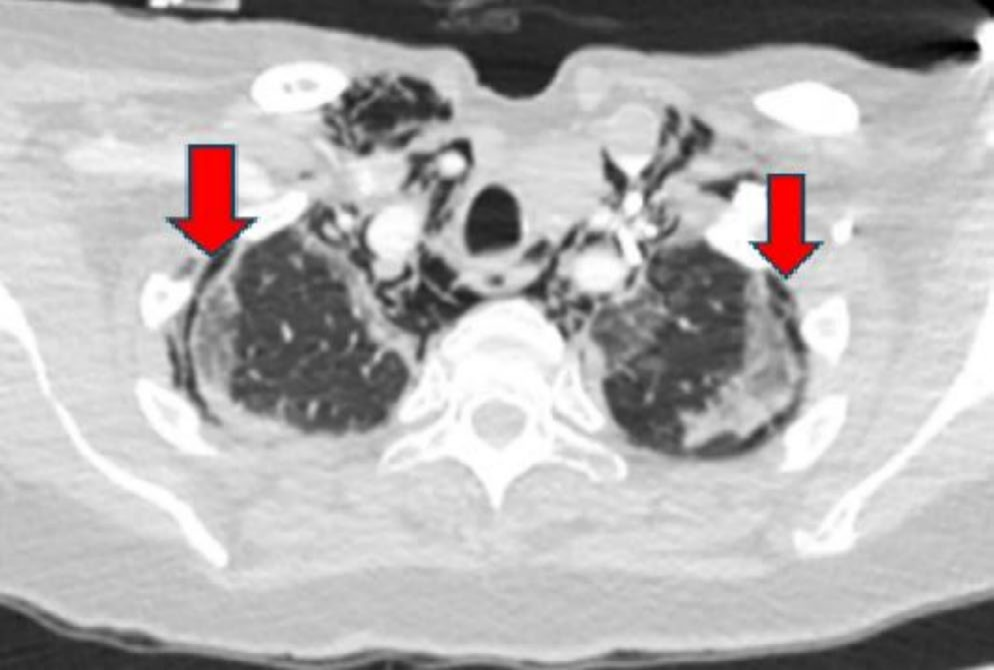
Chest scan demonstrating significant bilateral apical pneumothoraces. The red arrows are pointing to the areas of pneumothoraces on the CT chest scan.

The patient was again readmitted 2 weeks after discharge with worsening shortness of breath, over the preceding 24 h. A repeat CTPA was requested to exclude any new pulmonary embolism because of her progressively poor mobility since discharge. The CT scan showed extensive pneumopericardium (Figure 2A), along with pneumomediastinum (Figure 2B) and bilateral pneumothoraces (Figure 3). There were worsening areas of *bilateral basal peripheral and perilobular consolidations* with patchy areas of ground‐glass changes bilaterally. She was treated again with IV Meropenem and Clarithromycin for this episode. She also reported new onset dysphagia. She did not have noninvasive ventilation but had high‐flow oxygen to support her respiratory effort. She was also not escalated to the intensive care unit for invasive ventilation but she was managed in a high dependency unit.

**FIGURE 4:**
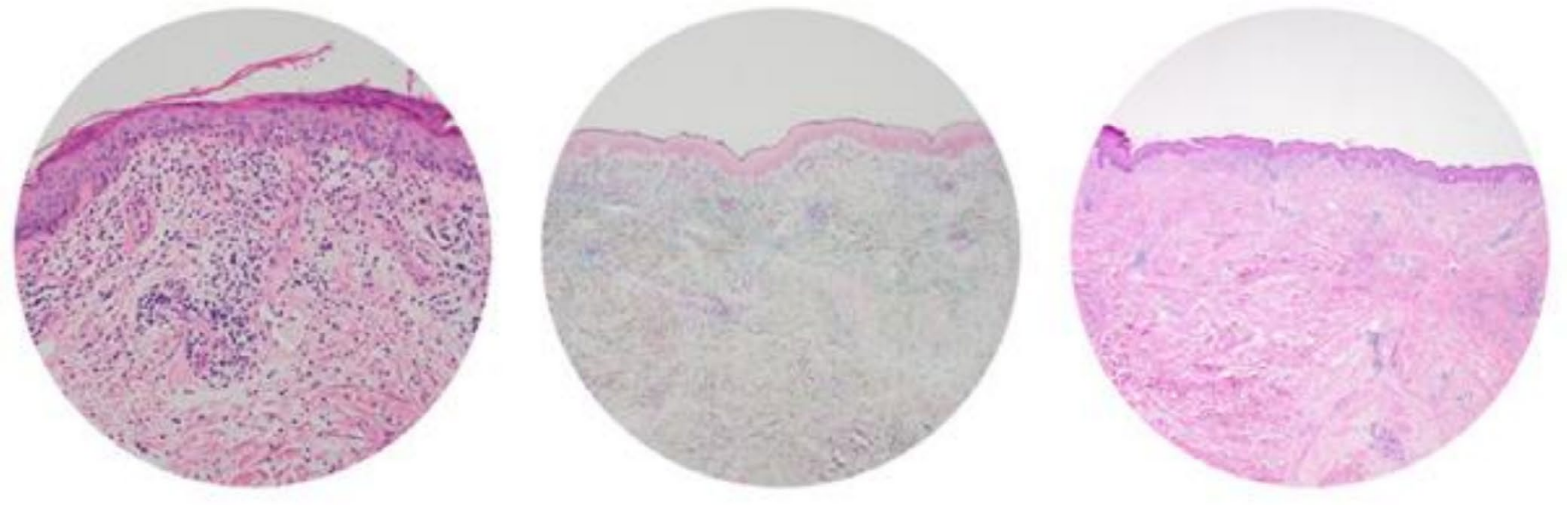
Skin biopsy from the skin around the right hip area demonstrating interface dermatitis compatible with dermatomyositis.

The rheumatology team reviewed her again and started high‐dose methylprednisolone 1 g daily for 3 days and intravenous immunoglobulins (IVIG). The patient died 2 months after her initial hospital admission due to rapid decline from haemodynamic compromise in spite of intensive immunosuppressant therapy. Her skin biopsy results (Figure 4) after her death confirmed dermatomyositis with interface dermatitis consistent with dermatomyositis. Her myositis antibody screening was positive for anti‐MDA5 antibodies and anti‐Ro‐52 antibodies (of uncertain significance). A list of all the tests and results she had with the timeline is shown in the Appendix A (Table A1).

## 3 Discussion

Few cases in the literature describe this triad of pneumomediastinum, pneumopericardium, and pneumothoraces in anti‐MDA5 dermatomyositis, making our report a valuable contribution to the clinical understanding of this unique diagnosis. Anti‐MDA5 dermatomyositis is associated with rapidly progressive ILD and skin manifestations, often with little to no muscle involvement [1] similar to the features seen in the presented case. The subgroup termed amyopathic dermatomyositis prior to the discovery of the anti‐MDA5 antibodies likely consists of a large proportion of MDA5‐positive patients [4]. The condition is thought to be triggered by viral infections, in part due to the presence of anti‐MDA5 in individuals recently diagnosed with SARS‐CoV‐2 infection, as well as the similarity in the pattern of lung disease seen in both conditions [5, 6]. However, no viral trigger was implicated in this case.

Anti‐MDA5 dermatomyositis can typically present with skin ulceration, which often forms punched‐out lesions over joints, especially the metacarpophalangeal and proximal interphalangeal joints of the hands. This is highly predictive of ILD development. Non‐scarring alopecia can also occur, making it important to differentiate this condition from systemic lupus erythematosus. Palmar papules, calcinosis, and more classical skin manifestations of dermatomyositis, such as Gottron's papules, shawl sign, heliotrope rash, mechanics hands, and Holster sign are also seen [2]. The case highlighted had skin manifestations but no skin ulcerations.

Rapidly progressive interstitial lung disease in MDA‐5 disease represents one of the respiratory features that contribute to poor prognosis in MDA‐5 disease. It responds poorly to treatment and is the leading cause of mortality. There is a 20 times higher risk of ILD in MDA‐5 positive disease when compared with other forms of dermatomyositis [7]. Radiologically, it can present with basal peripheral and perilobular consolidations with an organizing pneumonia pattern on HRCT, or with nonspecific interstitial pneumonitis (NSIP). This can rapidly progress to widespread consolidation of both lungs [8].

Another poor prognostic feature is pneumomediastinum. This occurs in roughly 10% of anti‐MDA5 positive patients. The median time to development is typically 5.5 months from diagnosis [9]. Pneumomediastinum refers to the presence of air within the mediastinal structures and is considered a rare but grave complication in the context of anti‐MDA5 DM associated with ILD. This occurs typically as a result of alveolar rupture secondary to inflammation‐induced fragility of the pulmonary interstitium. The Macklin effect describes the pathophysiological mechanism. Alveolar air dissects through bronchovascular sheaths toward the mediastinum, triggered by increased intrathoracic pressure or alveolar‐capillary barrier breakdown due to intense inflammatory activity [9].

The inflamed and structurally weakened alveolar‐capillary barrier in MDA5‐positive patients with ILD makes these patients particularly susceptible, often without any antecedent trauma or history of mechanical ventilation [9]. While pneumomediastinum can occur in other inflammatory lung diseases, its presence in anti‐MDA5 DM is strongly associated with high mortality, particularly when it arises early in the disease course, as seen in this case presented. Invasive mechanical ventilation should be avoided if possible, as it can exacerbate alveolar injury and hasten the spread of free air [10]. Non‐invasive ventilation has also been associated with worsening outcomes in this setting [10]. Therefore, oxygen therapy via low‐flow nasal cannula or high‐flow oxygen, aimed at promoting nitrogen washout and reabsorption of mediastinal air, remains the mainstay of respiratory support.

The onset of pneumomediastinum should prompt urgent reassessment of the immunosuppressive strategy and escalation of therapy, given its role as a surrogate marker of inadequate disease control [3, 9].

Two cohort studies analyzing Asian patients with anti‐MDA5 DM and the development of pneumomediastinum showed a 1‐year mortality of 53.3% and 60% [10, 11]. The presence of pneumomediastinum itself in isolation may not be a poor prognostic marker; however, in MDA5‐positive patients, they have a worse prognosis with pneumomediastinum when compared to patients with pneumomediastinum without anti‐MDA5 dermatomyositis [9]. In anti‐MDA5 dermatomyositis ILD, spontaneous pneumomediastinum and subcutaneous emphysema are common air‐leak complications [10]. On high resolution CTs (HRCT), most cases show the Macklin effect. That Macklin process explains the presence of pneumomediastinum. If the interstitial air then breaches the pleura, a pneumothorax can result as well. So the Macklin effect can precede or coexist with the pneumothorax, but it is not required for every pneumothorax present [12].

Another significant complication of MDA5 positive patients with ILD is pneumopericardium, which was also seen in this case. This is the presence of free air in the pericardial sac. This is even less commonly reported. It likely represents an extension of mediastinal air through pericardial reflections, again facilitated by inflammation‐compromised anatomical barriers. Its appearance, especially when coupled with pneumothorax and progressive consolidation, suggests extensive alveolar rupture and advanced disease. While sometimes incidentally detected, it is worth noting that pneumopericardium poses a direct risk of cardiac tamponade and sudden haemodynamic collapse. When tension physiology ensues, as reported by Okabayashi et al. [3], it can rapidly lead to sudden hemodynamic compromise. The case presented had a normal echocardiogram with no evidence of any cardiac tamponade.

In patients with underlying RP‐ILD, compromised pulmonary compliance can obscure early symptoms, and haemodynamic deterioration may evolve rapidly within hours especially if tension physiology develops [3]. Early recognition through imaging is essential. Management prioritizes conservative respiratory support with avoidance of positive pressure ventilation, as both invasive and non‐invasive mechanical ventilation can exacerbate pericardial air accumulation [10]. Serial echocardiographic monitoring is advisable in symptomatic patients or if tamponade is suspected. In this setting, pericardiocentesis may be lifesaving but is technically challenging due to the compressive air. The emergence of pneumopericardium should be viewed as a harbinger of critical disease progression in anti‐MDA5 DM and warrants immediate multidisciplinary review and escalation to maximal immunosuppressive therapy [3, 10].

Other clinical factors associated with a poor prognosis also include increasing age, rapidity of onset of respiratory symptoms within 3 months of presentation, dyspnoea, male gender, and fever at presentation. Clinically amyopathic dermatomyositis was also associated with poorer outcomes. A large proportion of these clinical risk factors are associated with the natural history of anti‐MDA5 dermatomyositis, and therefore the effect on prognosis may simply be due to the significant mortality associated with anti‐MDA5 dermatomyositis itself [13].

Apart from these clinical indicators of poor prognosis, biochemical factors have also been identified. The presence of Anti‐MDA5 antibodies itself has been found to be a strong predictive factor for mortality in Idiopathic Inflammatory Myopathy Interstitial Lung Disease (IIM‐ILD) [14]. High MDA5 titres at diagnosis may also be linked to more severe disease and poorer prognosis [15]. A high neutrophil to lymphocyte ratio in addition to peripheral lymphopenia is also associated with an increased risk of patient mortality. Elevated markers of macrophage activity, particularly ferritin, have also been linked with worse outcomes [13]. Both Interleukin‐6 (IL‐6) and Interleukin‐5 (IL‐5) have been studied and have both been found to be higher in patients with ILD but are more likely linked with disease severity rather than prognosis [16, 17].

British Society of Rheumatology (BSR) guidelines recommend that high‐dose corticosteroids should be considered as part of the induction regimen for treating RP‐ILD. Ciclosporin, tacrolimus, Rituximab, and cyclophosphamide can also be considered as part of this regimen. Corticosteroids can then be used with or without a concurrent conventional synthetic disease‐modifying therapy such as mycophenolate or azathioprine for chronic IIM‐ILD. This management should be carried out alongside an ILD‐specialist respiratory physician [18].

When evaluating the effectiveness of treating anti‐MDA5 dermatomyositis, one Japanese study found that initiating combined therapy with tacrolimus, cyclophosphamide, and high‐dose glucocorticoids achieved a higher 6‐month survival rate than step‐up therapy [19]. Rituximab has also been shown to induce remission in multiple case reports; however, its association with a higher risk of SARS‐CoV‐2 infection must be balanced against the benefit, as patients can present with both pathologies [20]. Intravenous immunoglobulin (IVIG) has been shown to be beneficial in cases of refractory dermatomyositis, and a retrospective study has shown similar results when comparing 6‐month mortality rates between patients who did and did not receive IVIG for anti‐MDA5 dermatomyositis [21, 22]. JAK inhibition therapies have also been studied for anti‐MDA5 dermatomyositis and have been shown to be an effective treatment for skin and ILD manifestations [23]. Plasma exchange may also have benefit in severe cases, but more evidence is needed for this therapy [24].

## 4 Conclusion

This case highlights several critical learning points. Pneumomediastinum, pneumothraces, and pneumopericardium in anti‐MDA5 DM are not merely radiological curiosities but harbingers of poor outcomes. Early recognition of anti‐MDA5 phenotypes, even before confirmatory serology, is crucial and should prompt aggressive upfront therapy. Multidisciplinary management with respiratory input, radiologic surveillance, and rheumatology oversight is vital, particularly in rapidly evolving respiratory compromise. Broader awareness and preparedness to escalate care with second‐line immunosuppressants, biologics, or novel agents are required. A strategic diagnostic and therapeutic algorithm that integrates serological, radiological, and clinical predictors while appreciating the nuances of anti‐MDA5 DM complications like air‐leak syndromes may improve survival in this otherwise devastating condition.

## Author Contributions

**Thomas Bond:** conceptualization, data curation, formal analysis, resources, software, writing – original draft, writing – review and editing. **Karan Kanani:** investigation, methodology, resources, software, validation, visualization, writing – original draft. **Mili Pansuria:** data curation, investigation, methodology, project administration, resources, software, validation, visualization, writing – original draft. **Kehinde Sunmboye:** conceptualization, data curation, investigation, project administration, software, supervision, validation, visualization, writing – review and editing.

## Consent

Written informed consent was obtained from the patient for the publication of this case report.

## Conflicts of Interest

The authors declare no conflicts of interest.

## Data Availability

The data underlying this case report are drawn with consent from the patient's clinical records held within the NHS Trust's electronic health record system. Due to the identifiable nature of these data and the conditions of ethical approval and patient confidentiality, they are not publicly available. Requests for access to anonymized data may be considered on a case‐by‐case basis and require approval from the relevant NHS Research and Development Department and Caldicott Guardian.

## Appendix A

**TABLE A1:** Blood results across the patients three hospital admissions.

Blood test	October presentation	November presentation	December presentation
Hb	127	118	116
WCC	4.3	7.1	15.6
Neutrophils	2.69	6.1	14.96
Lymphocytes	1.27	0.73	0.4
Platelets	305	333	415
eGFR	86	90	71
ALT	18	29	20
CK		85	62
CRP		< 5	32
ENA		Negative	
CCP		Negative	
RhF		Negative	
ANCA		Negative	
C3		0.91	
C4		0.26	
Immunoglobulin		Normal (no paraprotein)	
Blood cultures			Negative
Blood borne virus screen		Negative	Negative
Influenzae screen		Negative	
Pneumonia PCR panel			Negative
Urine culture			Negative
Sputum culture			Negative
Procalcitonin			0.5
